# Interpretation of metabolic memory phenomenon using a physiological systems model: What drives oxidative stress following glucose normalization?

**DOI:** 10.1371/journal.pone.0171781

**Published:** 2017-02-08

**Authors:** Veronika Voronova, Kirill Zhudenkov, Gabriel Helmlinger, Kirill Peskov

**Affiliations:** 1 M&S decisions LLC, Moscow, Russia; 2 Quantitative Clinical Pharmacology, Early Clinical Development, Innovative Medicines, AstraZeneca Pharmaceuticals, Boston, Massachusetts, United States of America; Stellenbosch University, SOUTH AFRICA

## Abstract

Hyperglycemia is generally associated with oxidative stress, which plays a key role in diabetes-related complications. A complex, quantitative relationship has been established between glucose levels and oxidative stress, both *in vitro* and *in vivo*. For example, oxidative stress is known to persist after glucose normalization, a phenomenon described as metabolic memory. Also, uncontrolled glucose levels appear to be more detrimental to patients with diabetes (non-constant glucose levels) *vs*. patients with high, constant glucose levels. The objective of the current study was to delineate the mechanisms underlying such behaviors, using a mechanistic physiological systems modeling approach that captures and integrates essential underlying pathophysiological processes. The proposed model was based on a system of ordinary differential equations. It describes the interplay between reactive oxygen species production potential (ROS), ROS-induced cell alterations, and subsequent adaptation mechanisms. Model parameters were calibrated using different sources of experimental information, including ROS production in cell cultures exposed to various concentration profiles of constant and oscillating glucose levels. The model adequately reproduced the ROS excess generation after glucose normalization. Such behavior appeared to be driven by positive feedback regulations between ROS and ROS-induced cell alterations. The further oxidative stress-related detrimental effect as induced by unstable glucose levels can be explained by inability of cells to adapt to dynamic environment. Cell adaptation to instable high glucose declines during glucose normalization phases, and further glucose increase promotes similar or higher oxidative stress. In contrast, gradual ROS production potential decrease, driven by adaptation, is observed in cells exposed to constant high glucose.

## Introduction

Diabetes mellitus is associated with numerous complications as well as an increased incidence of cardiovascular diseases (CV), resulting in a significant decrease in life quality and life span. For example, diabetic retinopathy is the most common cause of blindness; diabetic nephropathy is the leading cause of end-stage renal disease [1,2]. Increased blood glucose levels, or hyperglycemia, are known to be the main driving force underlying diabetic complications. In healthy subjects, daily blood glucose fluctuates within narrow intervals, whereas in diabetic patients, blood glucose level is, on average, higher and may also oscillate over a wide range, due to impaired glucose consumption within the circulatory system. Insulin replacement therapy and oral glucose-lowering agents, in combination with appropriate diet and life-style adaptations, may decrease blood glucose to near-normal values and significantly reduce the risk of complications [1].

Nevertheless, the importance of the initial glucose level control has been demonstrated in numerous animal and human studies [3]. In the UK Prospective Diabetes Study (UKPDS), the positive impact of intensive CV complication treatment was preserved during a post-treatment follow-up period, when patients received conventional therapy [4]. Conversely, in diabetic rats, poor glucose control led to hyperglycemia-induced changes in retinal cell apoptotic marker expression, which were sustained for as long as several months following glucose normalization [5]. Such a cellular imprint of the glycemic environment is now referred to as the “metabolic memory” phenomenon [6].

The objective of this modeling study was to analyze various experimental data on ROS generation in response to hyperglycemia measured *in vitro*, in order to provide a quantitative, mechanistic basis for this metabolic memory phenomenon. Evaluation of dynamics within key elements of this pathophysiological system, using a mechanistic physiological systems model, allowed us to characterize features of the phenomenon and to determine conditions leading to the development of metabolic memory.

## Materials and methods

### Model development

The phenomenon of metabolic memory has been actively studied in recent years [7]. General aspects of the potential underlying mechanisms have been hypothesized and studied, preferably through various *in vitro* experimental systems. Consequently, an increased generation of reactive oxygen species (ROS) in cellular respiratory chains, also known as oxidative stress, has been shown to be the main link between hyperglycemia and cellular malfunctions [7,8]. For example, oxidative stress can play a critical role in DNA methylation, causing the overexpression of proteins responsible for diabetic abnormalities, such as vascular endothelial growth factor (VEGF) and transforming growth factor (TGF) [9,10]. To capture and integrate our current knowledge around the metabolic memory phenomenon, several experimental facts need to be taken into account during the development and testing of a mechanistic, dynamic model.

First, several studies have demonstrated that despite short ROS half-lives, ROS production is maintained in excess for long periods of time after (and despite) glucose normalization [7,11]. These data may be related to ROS-induced DNA damage and epigenetic changes, which may lead to abnormal functioning of the intracellular respiratory chain and further acceleration of ROS production [6]. This positive feedback loop between DNA modifications and oxidative stress is considered to be a key driver of metabolic memory effect [6].

Second, there is substantial knowledge on adaptive potential and changes, within this glucose regulatory system. For instance, ROS generation can be stabilized and even decreased, given continued (constant) high glucose exposure. This observation may be explained by adaptive mechanisms, which protect cells from excessive oxidative stress exposure [7]. Interestingly, it was also shown that cells exposed to oscillatory glucose levels produce higher ROS levels *vs*. cells exposed to a time-equivalent, albeit constant glucose concentration, thereby pointing to the complex nature of this adaptive processes [9,12–14].

**Fig 1:**
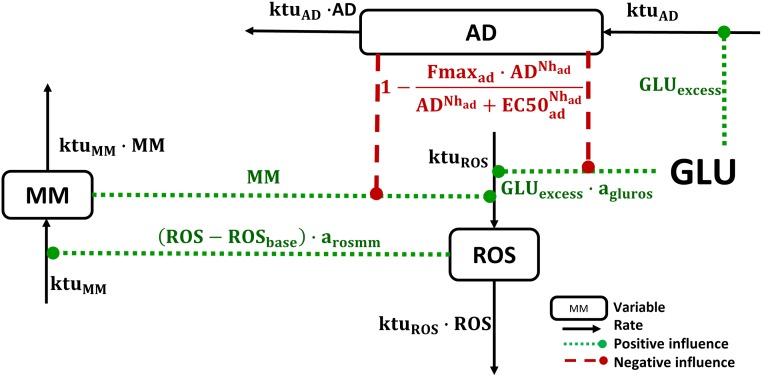
Model schematic. Glucose stimulates ROS production (**ROS**) and additionally promotes cellular adaptive processes (**AD**)—the latter then mitigates further glucose-dependent ROS generation and subsequently allows for the development of metabolic memory (**MM**). ROS and MM positively affect each other, whereas AD is stimulated by glucose excess and negatively influences ROS synthesis. Boxes denote model variables, black arrows denote reaction rates, dotted lines denote positive influences, and dashed lines denote negative influences.

Based on these experimental observations, we here propose a physiological systems model with three variables, interconnected through: (a) a positive feedback loop (ROS levels *vs*. metabolic memory development), and (b) a negative feedback loop (ROS generation *vs*. cellular adaptive processes) (Fig 1). The proposed variables are:

1. “ROS”, which represents a set of complex functions, which describes oxidative damage being accumulated during cell exposure to high glucose. It should be mentioned, that the model describes the steady-state ROS concentration that was measured in the experiments after several hours at the constant glucose level. Thus, this work is not aimed at reproducing the short-term dynamics of ROS levels. Therefore, in the model the ROS variable does not fully reflect the time behavior of real ROS, though it coincides with measured values of ROS in considered experimental studies [12,13,15–21]. Despite rapid ROS turnover, gradual ROS increase in response to hyperglycemia *in vitro* is observed [15]. Therefore, we assumed ROS levels to be determined by some metabolites with slower half-lives (on the order of several hours), which can be characterized as “ROS production potential”. Since ROS turnover is fast, its dynamics reflects the dynamics of this potential. There are no available experimental data to quantify ROS dependence on ROS production potential and we cannot differentiate between these two variables in the model. To preserve model identifiability, we thus used a single “ROS” variable in the model. Hyperglycemia and metabolic memory promote excessive ROS production, whereas cellular adaptive processes decrease detrimental ROS effects on cells.
2. “MM”, which represents metabolic memory–an accumulation of ROS-related cell abnormalities, *e*.*g*., damage along the respiratory chain and other mitochondrial malfunctions. MM dynamics are determined by a zero-order synthesis rate and a first-order elimination rate. MM synthesis is triggered by an initial generation of oxidative stress species.
3. “AD”, which represents cellular adaptive processes. These processes mitigate detrimental glucose effects upon the generation of oxidative stress species. Hyperglycemia triggers activation of AD, which in turn may inhibit ROS synthesis both *via* (a) direct glucose and (b) MM-related effects. A Hill equation was used for the description of these negative feedback effects.

Additional model assumptions were considered, to adequately describe available experimental data and to set physiologically-based initial conditions:

1. Glucose concentration (GLU) was set as either (i) a constant parameter, for experimental conditions where constant glucose exposure was used, or (ii) an explicit time-varying driving function, when oscillatory glucose conditions were used. Additionally, the following parametrization was used to describe detrimental variations in glucose levels, according to the study design:
  $$\text{if}\left(\text{GLU}>{\text{GLU}}_{\text{basal}}\right)\;{\text{then GLU}}_{\text{excess}}=\text{GLU}-{\text{GLU}}_{\text{basal}}\;{\text{else GLU}}_{\text{excess}}=0$$
2. The initial value of ROS in the healthy state was set to 1 and this then represents a state, whereby ROS generation is well controlled by tissues not stimulating metabolic memory or cellular adaptive processes development:
  $$\text{if}\left(\text{ROS}>{\text{ROS}}_{\text{basal}}\right){\text{ then ROS}}_{\text{excess}}=\text{ROS}-{\text{ROS}}_{\text{basal}}{\text{else ROS}}_{\text{excess}}=0$$
3. A linear dependence between glucose level and ROS production rate was proposed, based on the experimentally established correlation between glucose concentrations and observed ROS levels [21].
4. The main data source used here for the estimation of model parameters was derived from glucose stimulation of HUVEC cells *in vitro* [12,13,15–21]. For such *in vitro* conditions, we assumed ROS generation to be maintained at a steady-state level, following glucose normalization. Though this may differ *in vivo*: for example, ROS excess may be removed from tissue via bloodstream clearance, thereby breaking this steady-state assumption.

The proposed model is represented by a system of ordinary differential equations (ODE), which describe the dynamics of key system components (Eq 1):

$$\{\begin{array}{c} \frac{\text{dMM}}{\text{dt}}={\text{ktu}}_{\text{MM}}\cdot \left(\left({\text{ROS}}_{\text{excess}}\right)\cdot {\text{a}}_{\text{rosmm}}-\text{MM}\right)\\ \frac{\text{dAD}}{\text{dt}}={\text{ktu}}_{\text{AD}}\cdot \left({\text{GLU}}_{\text{excess}}-\text{AD}\right)\\ \frac{\text{dROS}}{\text{dt}}={\text{ktu}}_{\text{ROS}}\cdot \left(\left(1+\left({\text{GLU}}_{\text{excess}}\cdot {\text{a}}_{\text{gluros}}+\text{MM}\right)\cdot {\text{AD}}_{\text{ef}}\right)-\text{ROS}\right)\\ {\text{AD}}_{\text{ef}}=1-\frac{{\text{Fmax}}_{\text{ad}}\cdot {\text{AD}}^{{\text{Nh}}_{\text{ad}}}}{{\text{AD}}^{{\text{Nh}}_{\text{ad}}}+\text{EC}50_{\text{ad}}^{{\text{Nh}}_{\text{ad}}}} \end{array}$$ (1)

where MM, AD and ROS are model variables corresponding to metabolic memory, cellular adaptive processes, and ROS generation; AD_ef_ is the effective value of ROS generation decrease by cellular adaptive processes; ktu_MM_, ktu_AD_, ktu_ROS_ are turnover constants for variables MM, AD, and ROS, respectively; a_rosmm_ and a_gluros_ are parameters describing positive feedbacks between ROS and MM; Fmax_ad_, $\text{EC}50_{\text{ad}}^{{\text{Nh}}_{\text{ad}}}$, Nh_ad_ are the parameter set for adaptive processes.

Several literature data sources were used for model calibration [12,13,15–21]. Seven model parameters describing ROS turnover and cellular adaptive processes were estimated based on *in vitro* ROS production data. For this purpose, 43 experimental data points from 9 published studies were collected and combined into a pooled dataset. Similarity in experimental design was a key study inclusion criterion. Specifically, experimental data were included if:

1. Studies were performed on HUVEC cultures;
2. ROS production was evaluated using a fluorescence assay or *via* measurement of 8-hydroxydeoxyguanosine (8-OHdG), as described in [20,21];

ROS levels, in the experiments, were normalized by control ROS conditions (normoglycemia); this allowed for partial reduction of inter-study variability.

Additionally, the model was required to reproduce two main experimental settings with different glucose exposure regimens: one regimen with constant high glucose (CG); one regimen with oscillatory glucose, between normal and high levels, over fixed time intervals (OG). In most of these experiments, ROS level was measured either during CG/OG exposure or after glucose reaching a normal steady-state level (NG).

All model parameters and estimation methods are summarized in Table 1.

**Table 1:** Values of the model parameters.

Parameter	Description	Value	RSE^1^, %	Dimension	Estimation method
GLU_basal_	Maximum glucose level, healthy state	9	-	mM	Fixed—based on data published in [22]
ROS_basal_	Normalized basal ROS level in experiments	1	-	dimensionless	Based on assumption “a” described in the *Model Development* paragraph
ktu_MM_	MM elimination constant	0.007	-	1/hour	Calculated from mitochondrial protein half-life (equal to 4 days [23]).
a_rosmm_	Linear ROS effect on MM synthesis	1	-	dimensionless	Based on assumption “d” described in the *Model Development* paragraph. See also table footnote^2^
Fmax_ad_	Maximum AD effect on ROS synthesis	0.8	-	dimensionless	Fixed—according to expression data of proteins responsible for adaptation to oxidative stress (*e*.*g*., TIGAR, MDM-2, etc.) [7]
Nh_ad_	Hill coefficient for adaptation to ROS synthesis	5	-	dimensionless	An approximate estimate (based on the number of fixed value runs, see^3^)
ktu_ROS_	ROS elimination constant	0.0316	53.05	1/hour	Estimated according to data published in [12,13,15–21]
ktu_AD_	AD elimination constant	0.00714	1.41	1/hour	
a_gluros_	Linear glucose effect on ROS synthesis	0.364	31.36	1/mM	
EC50_ad_	EC_50_ for AD effect (equation relating AD effect to ROS synthesis)	6.142	0.64	-	
Ω for ktu_ROS_	Inter-study variance for ROS elimination^4^	1.145	33.76	1/hour	
Ω for a_gluros_	Inter-study variance for glucose effect on ROS synthesis^4^	0.936	22.79	1/mM	
b	Proportional residual error^5^	0.09932	18.65	%	

^1^Relative standard error.

^2^Parameter a_rosmm_ determines system behavior after glucose normalization. Depending on parameter a_rosmm_ value, ROS production potential may either decrease to normal values, remain at steady-state, or accumulate. In accordance with the definition of metabolic memory, abnormal ROS production is held constant after glucose decrease (6). Thus, for reaching the steady-state conditions, parameter a_rosmm_ can be expressed using the following equation:
$${\text{a}}_{\text{rosmm}}=\frac{\text{ROSss}-1}{\text{ROSss}-{\text{ROS}}_{\text{basal}}},$$
where ROS_ss_ is the steady-state ROS level after glucose normalization.

^3^The following approach was used for the Hill coefficient estimation. The fixed value of this parameter was varied over a range of 0.1 to 10. An analysis of parameter estimation outcomes (likelihood value and RSE of estimated parameters) demonstrated that the goodness-of-fit improved with Hill coefficient increase and starting from the value of five and above the model produced the same goodness-of-fit. This value makes physiological sense, considering that these cellular adaptive processes tend to exhibit a switch-like behavior, with a maximal level being rapidly reached after an initial glucose stimulation [7].

^4^The rate of ROS production potential change as well as overall ROS concentrations in response to a given glucose stimulation do show high inter-study variability, even when considering comparable experimental settings across the literature references which were used. A non-linear mixed effects (NLME) approach was used to adequately quantify inter-study variability. Based on parameter estimation results and the goodness-of-fit analysis, two random effects were introduced into the model, namely on kel_ROS_ and a_gluros_:
$$y_{j}=f_{j}+{\text{ε}}_{j},$$
$$\;where\;f_{j}=f\left(k_{j}^{1},k_{j}^{2},\ldots ,\;k_{j}^{i}\right)$$
Function *f* describes the model structure; $k_{j}^{i}$ parameters represent population parameters including kel_ROSj_ and a_glurosj_ for j^th^ subject; ε_*j*_ is the residual error.

^5^Several residual error models were tested, including constant, proportional and different combined error types. The proportional error model was identified as the best one given the data:
$${\text{ε}}_{j}=b*f_{j}*e_{j},$$
where b is a coefficient, e_j_ is a random number.

### Software

Model development and analysis was performed using the IQM toolbox [http://www.intiquan.com/iqm-tools] for MATLAB 2013b. Visualization of the obtained simulations was performed in R software version 3.2.5 using the ggplot2 2.1.0 package and the plot3D 1.1 package. NLME model analysis and parameter estimation was based on the Stochastic Approximation Expectation Maximization (SAEM) algorithm and performed using the Monolix software [http://monolix.lixoft.com]. Model quality was evaluated using the following criteria: change in the objective function value (logarithm of likelihood, Akaike information criterion), inspection of diagnostic plots, precision of parameter estimates (based on estimated RSE values), as well as minimization of inter-study and residual variability. The initial set of parameters for the model calibration was chosen based on physiological limits available from literature sources. Based on such limits, 100 sets of physiologically-plausible initial parameter values were randomly generated, and parameter estimation was, for each initial value set, performed. Upon completion of this procedure, the estimated set of parameters values was collected. It was unique and did not depend on initial values, which in turn also supports model identifiability.

Local sensitivity analysis of model parameters was performed based on the following algorithm:

1. Each model parameter was changed within a 50% range of the estimated population value, in uniform, incremental steps.
2. ROS production by cells exposed to 20 mM of CG conditions during two weeks was predicted using estimated and changed from estimated parameter values reported in Table 1.
3. Comparisons of predictions were performed at the 24-hour, 2-week, and 12-week time points, to compute relative ROS increase sensitivity values.
4. The impact of each parameter change on model predictions was ranked and displayed using tornado plots, as described previously [24].

## Results

### Analysis of model quality and predictions

**Fig 2:**
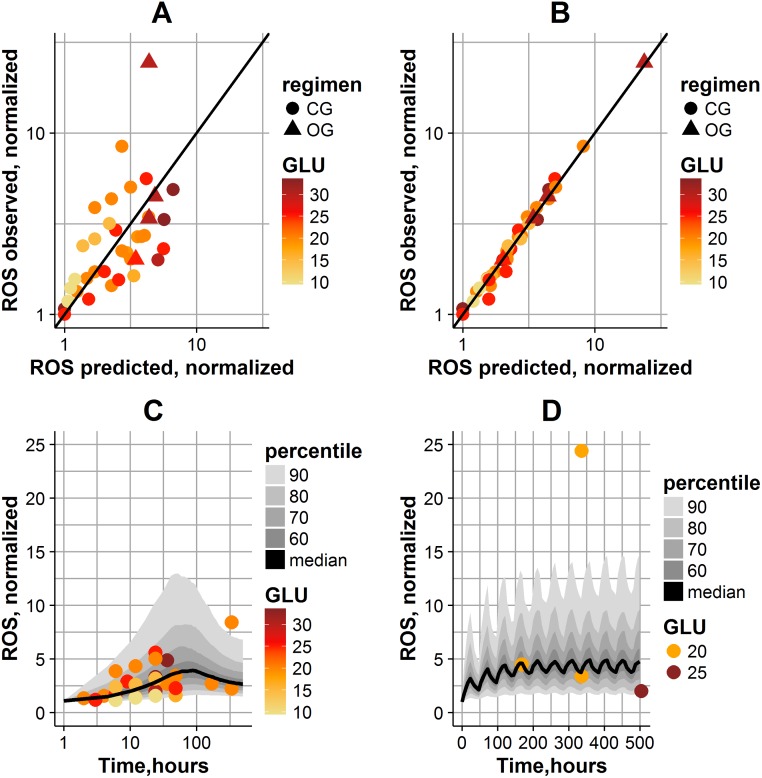
Model quality in reproducing data. (A) Observations *vs*. population model predictions. (B) Observations *vs*. individual model predictions. The straight line represents a perfect agreement between experimental and calculated values. The magnitude of glucose exposure is coded by color; the type of experiment is coded by dot shape. (C) Population simulations of ROS dynamics in CG experiments. (D) Population simulations of ROS dynamics in OG experiments. Solid line denotes model-predicted median; gray shades correspond to different percentiles of population predictions; the magnitude of glucose exposure is coded by color; the type of experiment is coded by dot shape.

Model quality in reproducing the data is shown in Fig 2. A high inter-study variability in ROS levels is observed. Consequently, population parameter values are able to reproduce average trends found in the data (Fig 2A). By taking into account the inter-study variability for two parameters, kel_ROS_ and a_gluros_ (respectively, glucose effect on ROS generation, and ROS turnover), the individual fits were significantly improved in the model (Fig 2B). Estimated RSEs of the parameters were relatively small, indicating the good precision in parameter estimates (Table 1). The quality of the model was also evaluated by simulating data using final parameter estimates (fixed and random effects) and assessing the visual predictive check. Based on simulation results, the model adequately reproduced ROS dynamics, for the experimental CG and OG exposure protocols used in cells *in vitro* (Fig 2C and 2D). The prediction interval captured all experimental data, except for two points, which were both observed in the same study: these outlier points may be explained by the specific experimental settings used in that study (oxidative stress was measured using 8-OHdG assay in this study) [13].

According to Fig 2, the model predicted the bell-shaped ROS dynamics in cells exposed to CG (Fig 2C), and oscillating ROS dynamics in cells exposed to OG (Fig 2D). It is interesting to note that ROS levels remained above normal values, even during NG periods within the OG regimen. Overall ROS levels were higher in OG-exposed cells, which is in agreement with the observation that OG exposure is more detrimental to cells than a CG exposure. After glucose normalization, the model predicted a decrease in ROS levels to 46–63% following glucose normalization. For an OG-NG exposure regimen, the model predicted similar ROS levels before *vs*. after glucose normalization, which is also in good agreement with comparable experimental data points found in the literature [7,11].

**Fig 3:**
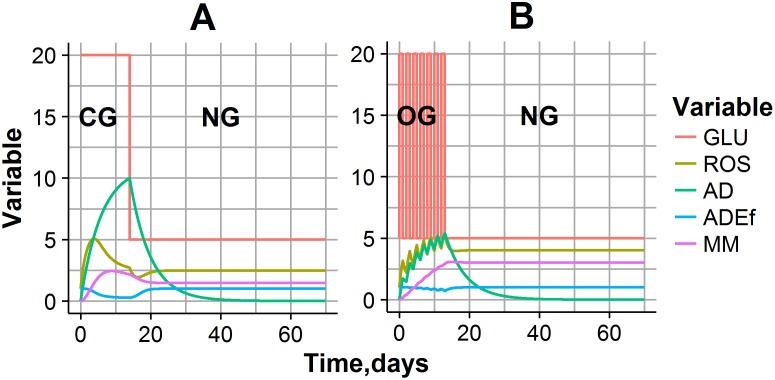
Predictions of model variables and their dynamics, for typical experimental settings. (A) CG exposure experiment: Glucose was maintained at 20 mM for 14 days, then was decreased to 5 mM. (B) OG exposure experiment: glucose was allowed to oscillate between 5 mM and 20 mM over 24-hour intervals for 14 days, then was decreased to 5 mM.

Simulation typical CG and OG experimental settings are shown in Fig 3. During CG exposure experiments, when glucose level is maintained at 20 mM for 14 days, bell-shaped ROS dynamics were observed (Fig 3A). This complex behavior can be interpreted by a cellular adaptive process: a gradual accumulation of AD during high glucose exposure decreases the glucose effect on ROS production, and consequently decreases oxidative stress. In fact, such adaptation exhibits a prolonged effect, even after NG conditions have set in, resulting in a temporal decline in ROS concentrations (Fig 3A). However, ROS excess during CG or OG exposure also allows for the gradual accumulation of MM, which, in turn, shifts the quasi steady-state level of ROS, typical for NG conditions, to a new, higher value. Oscillating ROS dynamics mirrored the oscillating glucose exposure in the OG experiment (Fig 3B). AD and MM accumulation was also observed in this simulated experiment, however the AD level at the end of OG exposure was lower *vs*. CG exposure. This may explain higher ROS and MM levels at the end of the OG exposure experiment, as compared to CG exposure, and provides evidence for more a detrimental effect following unstable (time-varying) glucose regimens, such as abnormal increase of cell proliferation, hyperactivation of p53 related to cell senescence etc. [7,9].

### Model simulations of glucose stimulatory effects, depending on various experimental designs

**Fig 4:**
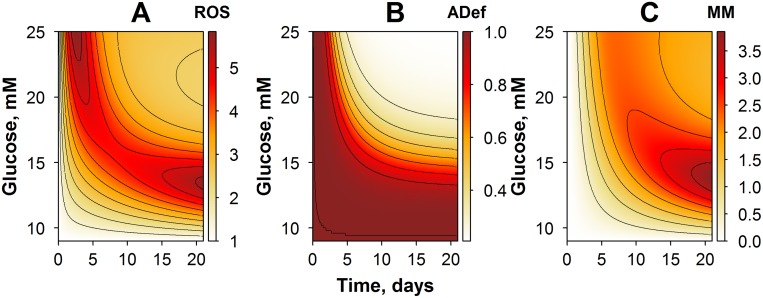
Contour plots of model simulations: variables and their dynamics in an experimental setting of CG exposure, with varying glucose amplitude. (A) Simulations of ROS dynamics. (B) Simulations of cellular adaptive processes. (C) Simulations of metabolic memory dynamics.

**Fig 5:**
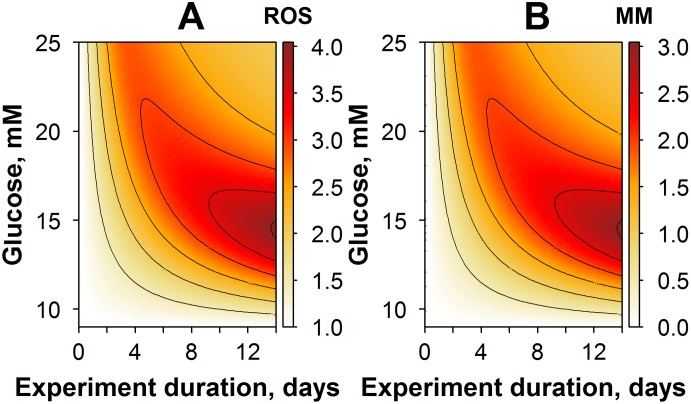
Contour plots of model simulations: variables and their steady-state levels, after reaching an NG exposure condition. The duration of cell exposure to high glucose and to glucose levels during the experiment was varied. (A) Simulations of ROS dynamics. (B) Simulations of metabolic memory dynamics.

**Fig 6:**
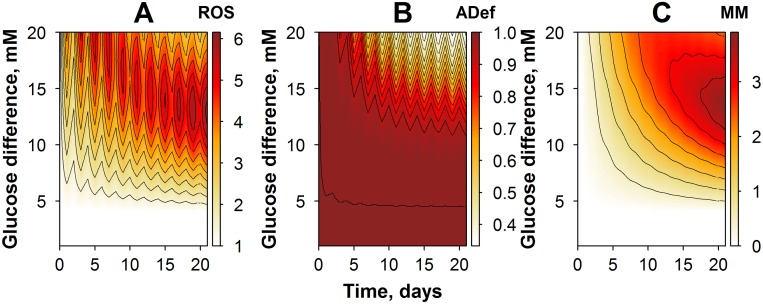
Contour plots of model simulations: variables and their dynamics, in an OG experimental setting with varying glucose amplitude. (A) Simulations of ROS dynamics. (B) Simulations of effective cellular adaptive processes. (C) Simulations of metabolic memory dynamics.

The proposed model and its corresponding simulations already highlighted, in the present glucose-ROS regulation system, a number of complex, nonlinear responses, which are dependent on both the amplitude and the duration of initial glucose exposure. One may next use the model to investigate changes in system behavior, according to various experimental protocols, which the user may want to explore *via* simulations. We thus simulated several experimental protocols, within a range of physiological conditions typical of diabetic patients, *e*.*g*., by varying glucose exposure amplitude and/or duration, during the glucose exposure phase. Simulation results were captured in contour plots pictured in Figs 4–6.

In the first study case, we predicted, *via* simulations, responses to an experimental setting with CG exposure (Fig 4). Glucose levels were allowed to vary over a 9- to to 25-mM range, with an overall CG exposure duration of 21 days. Model predictions demonstrated that the time to reach ROS level maxima is dependent on the amplitude of the glucose exposure (Fig 4A). A gradual accumulation of ROS production potential was observed when cells were exposed to glucose levels lower than 16 mM, whereas the bell-shaped ROS dynamics were obtained at higher glucose levels. In fact, this effect corresponded to a more rapid cellular adaptation in the higher glucose case (Fig 4B). Interestingly, dynamics of the MM variable, in general, followed the dynamics of ROS behavior, although the maximal effect on metabolic memory was achieved already with moderate glucose exposures, in the range of 12 to 16 mM (Fig 4C). These simulations demonstrated a tight relationship between MM and ROS, *via* a system of positive feedback mechanisms captured in the model. They provided further evidence that the cellular adaptive processes were more effective in conditions of higher glucose and longer duration in glucose exposure.

Another interesting model-based investigation is the study of how glucose amplitude and duration in the simulated experiments may affect ROS quasi steady-state levels achieved after reaching NG conditions. To this end, ROS levels were predicted for a set of various experimental settings. In particular, cells were exposed to constant high glucose levels ranging from 9 to 25 mM, with durations of one hour and up to two weeks (Fig 5). Overall, the key model variables exhibited response profiles, which were comparable to those predicted for CG exposure conditions. Thus, the longer the duration, or the higher the amplitude in glucose exposure, the higher the ROS production and the subsequent effect on metabolic memory accumulation. Typically, changes in the AD variable were not seen under NG exposure conditions, as adaptation (cellular adaptive processes) are strictly dependent upon non-NG conditions. However, the influence of such adaptive processes was important to explain differences in maximal ROS levels and MM responses, which can be observed in experiments with higher durations of glucose exposure.

Effects of oscillatory glucose exposures were of particular interest, since such conditions may actually reflect best the pathological state in diabetic patients. The following experimental design protocols were considered, in our simulations of OG exposure conditions: cells were exposed to OG with an amplitude of oscillations ranging from 0 to 20 mM (“Y” axis on Fig 6), and a corresponding maximal glucose level of 25 mM. Duration of the experiment was set to 21 days (Fig 6). As demonstrated in Fig 6A, ROS dynamics was affected by the amplitude of glucose oscillations. For example, OG conditions with an amplitude lower than 5 mM did not result in oxidative stress production, while higher OG amplitudes promoted a striking increase in ROS levels. ROS dynamics behavior, in turn, determines the dynamics of cellular adaptive process (adaptation), as shown in Fig 6B. In particular, lower amplitudes in OG were not sufficient to switch on the adaptation effect; only high or very high OG amplitudes were able to trigger a visible adaptation effect. This was in sharp contrast with CG exposure conditions (Fig 4), where even moderate levels of glucose exposure could initiate a prominent response in adaptation. Consequently, there was an increase in cumulative ROS levels due to higher MM accumulation under OG exposure conditions (Fig 6C).

### Local sensitivity analysis

**Fig 7:**
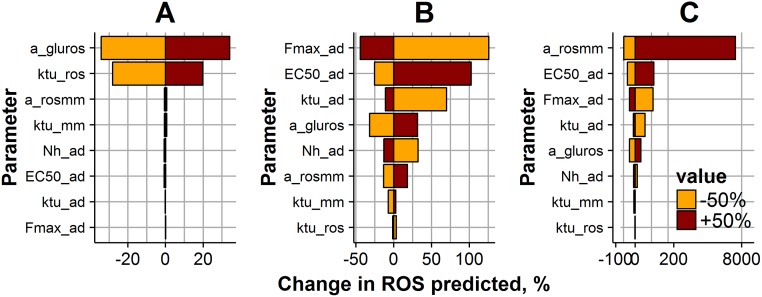
Impact of model parameters on ROS levels, during a 2-week, 20 mM CG exposure experiment. ROS levels were predicted at three different time points: the early ROS response at 24 hours after the start of the experiment (A); ROS levels at 2 weeks after the start of the experiment (B); ROS levels after achieving NG exposure conditions, 12 weeks after the start of the experiment (C). Parameter values were varied ± 50% (brown bar color—for positive change; orange color—for negative change) from the initial estimate (Table 1). Bar size and X-axis represent the magnitude of the parameter change effect on the ROS value.

**Fig 8:**
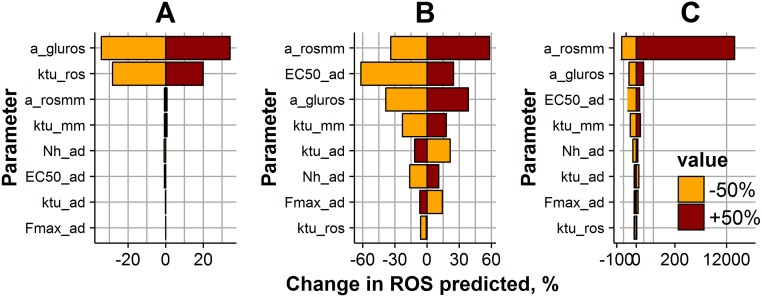
Impact of model parameters on ROS levels, during a 2-week, 5–20 mM OG exposure experiment. ROS levels were predicted at three different time points: the early ROS response at 24 hours after the start of the experiment (A); ROS levels at 2 weeks after the start of the experiment (B); ROS levels after achieving NG exposure conditions, 12 weeks after the start of the experiment (C). Parameter values were varied ± 50% (brown bar color—for positive change; orange color—for negative change) from the initial estimate (Table 1). Bar size and X-axis represent the magnitude of the parameter change effect on the ROS value.

The impact of specific parameter changes on predicted ROS levels and following glucose exposure was investigated using a local sensitivity analysis. A standard CG setting of 20 mM glucose exposure (Fig 7) or OG oscillations between 5 mM and 20 mM over 24-hour intervals (Fig 8) was considered for this sensitivity analysis, along with a duration of two weeks. Additionally, three characteristic time points were used: the time of initial response to glucose exposure, 24 hours following the start of the experiment (Figs 7 and 8A); the time of glucose exposure completion, two weeks after the start of the experiment (Figs 7 and 8B); and the time of return to NG exposure conditions, 12 weeks after the start of the experiment (Figs 7 and 8C).

Sensitivity profiles turned out to differ for the three different characteristic time points. In particular, ROS dynamics during the first day of the experiment were mainly driven by parameters a_gluros_ and ktu_ROS_, which are responsible for the direct glucose effect on the ROS production potential accumulation rate (Figs 7 and 8A). ROS production potential after two weeks of glucose exposure was significantly affected by any of parameter change (Figs 7 and 8B). It is interesting to note that different factors are responsible for ROS levels in cells exposed to CG and OG regimens during 2 weeks. Higher ROS dependence on parameters responsible for cellular adaptive processes, Fmax_ad_, ktu_ad_ and EC50_ad_ is observed for CG regimen (Fig 7B), whereas ROS level in cells exposed to an OG regimen depends on parameters arosmm, EC50_ad_ and a_gluros_ (Fig 8B). Interestingly, after reaching NG exposure conditions, the parameter most sensitive to ROS level changes for both CG and OG regimens was a_rosmm_, a parameter that is key in the development of metabolic memory (Figs 7 and 8C).

## Discussion

We developed a minimal mechanistic model that captures, in kinetic and quantitative terms, the effects of glucose on oxidative stress and on metabolic memory. We calibrated the model based on multiple sources of experimental data available from the published literature. The proposed model structure takes into account the key processes responsible for ROS generation under various profiles of hyperglycemic conditions. The model implementation and calibration approach combined the use of fixed parameters, in agreement with realistic physiological limits, and the NLME method, for the estimation of unknown parameter values, to better handle uncertainty around model-based predictions and to increase the model predictive power. This approach, in turn, also made it possible to build a model with a clear and interpretable structure, which may then be used to better understand mechanisms and conditions, which underlie detrimental, time-dependent glucose effects in healthy tissue and in diabetics. Thus, the calibrated model allowed us to explain patterns of ROS in response to various glucose exposure regimens, as tested *in vitro*, and to extrapolate such knowledge towards untested experimental protocols and conditions, such as in diabetics. As an example, the model was able to reproduce the observed “metabolic memory” phenomenon and to predict ROS excess as observed after glucose normalization conditions [7]. This behavior was described in the model by a minimal system of positive and negative feedback loops, between ROS generation and generic variables, which describe metabolic memory and the development of cellular adaptive processes (adaptation) (Fig 1).

Differences in the accumulation rates of the proposed model variables could account for the quantitative effects observed in *in vitro* experiments and were in good agreement with multiple experimental data reported in the literature. [23,25]. Such an experimental observation finds its basis through a most sensitive parameter identified in the model for NG exposure conditions, mainly responsible for the development of metabolic memory. Indeed, small changes in parameter a_rosmm_, which underlies the positive feedback loop between ROS excess and metabolic memory generation, can be sufficient to support increased ROS steady-state levels, post-glucose normalization. However, *in vitro* studies of glucose stimulation typically do not extend measurements for stability of ROS at steady-state, post-glucose stimulation. Unlike to *in vitro*, *in vivo* ROS dynamics is affected not only by its production and elimination but also by elution from the tissue. Therefore, the feedback loop between ROS and MM can be interrupted and ROS production can return to normal state. In the current model framework, the gradual ROS decrease to normal levels can be reproduced assuming a_rosmm_<1; however, the lack of quantitative data does not allow us to estimate the value of a_rosmm_, *i*.*e*., the rate of system recovery. This model limitation is a key point to consider for future development, *e*.*g*., towards predictions of the system’s behavior *in vivo*; to this end, incorporation of *in vivo* data on metabolic memory will be critical. Metabolic memory phenomenon was supported by preclinical studies, e.g., diabetic animals treated after periods of poor glycemic control [5]. Such studies, however, report only a single time point measurement of ROS levels following glucose normalization; this is in line with our model-based results, but would not allow us to estimate the dynamic trend of oxidative stress following normalization.

Another set of paradoxical experimental data were reproduced by the model, namely the more profound effects of oscillatory *vs*. constant glucose exposure settings, on ROS stimulation [7]. No robust hypothesis underlying the driving force between cellular adaptation and hyperglycemia has been reported to date in the literature. In the present model, however, the observed effect could be implemented *via* incorporation of a semi-mechanistic cellular adaptation process, directly triggered by a glucose excess. Adaptation is a function of the excess glucose exposure AUC, and the OG protocol simply does not lead to as much accumulation of AD because of less total exposure. The key to the difference between the CG and OG scenarios is the non-linear feedback from AD to ROS production, which is not activated until AD has reached a certain threshold—which is less likely to occur under OG stimulation. Moreover, according to the model simulations, OG exposure with low-amplitude oscillations would not cause oxidative stress. This model prediction is in good agreement with clinical data indeed: a low incidence of diabetic complications has been reported, in patients with tight glycemic control, although with plasma glucose levels still higher as compared to healthy subjects [26]. According to the American Diabetes Association (ADA) guideline, the targeted daily blood glucose level for non-insulin dependent diabetic patients is 4.4–10.0 mM [27]. This is in accordance with *in vitro* data, since oxidative stress is observed in cells exposed to 10 mM CG and higher [21]. Additionally, results from our sensitivity analysis (Figs 7B and 8B) indicated that model parameters responsible for the adaptation process most influence ROS levels after prolonged cell exposure to HG. All these findings confirm the importance of keeping daily blood glucose levels within a specific range: a narrow interval with a particular mean value of glucose level is more beneficial than a wider one with the same mean value. It points to the important benefits of, for example, dietary intervention associated with lower glucose supply from food intake and reduction of prandial glucose peaks, on top of glucose-lowering agents or insulin replacement therapy. Oscillatory glucose regimens can indeed cause the highest level of metabolic memory building-up, with a potential for subsequent diabetic complications (Fig 6C). This is further supported by reports showing that glycosylated hemoglobin levels (HbA1c), which correlate well with averaged plasma glucose levels, are not necessarily a good predictor for diabetic complication risk, whereas plasma glucose instability can be more important [28]. HbA1c has been, for years, a gold standard for metabolic control evaluation in diabetic patients [28]. However, today’s continuous glucose monitoring (CGM) systems do allow for daily blood glucose dynamics tracking, and would therefore allow for the development of a quantitative algorithm linking such glucose dynamics history to oxidative stress levels and risk of diabetic complications.

A current limitation in the present *in vitro* study on glucose-stimulated oxidative stress generation (from both experimental and modeling perspectives) lies in the absence of detailed, informative longitudinal data, which would profile the ‘metabolic memory’ phenomenon. Also, an accurate quantitative description of the *in vitro* data is highly limited by inter-study variability, which may obscure complex systems dynamics. In our modeling approach, the use of an NLME technique was of great help in the further analysis of pooled datasets from studies of comparable experimental conditions. This allowed us to handle data variability well and to identify a population set of parameters, to describe average trends in ROS dynamics over a continuous timescale. However, individual level data would be required, to gain further insights into variability source(s). Meanwhile, the developed model may be used as a tool for improved, informed experimental study design, which in turn may provide further understanding of glucose effects on oxidative stress.

## Conclusion

We developed a minimal, semi-mechanistic model that describes current data and hypotheses on glucose dynamical effects upon oxidative stress, as observed *in vitro*. The calibrated model predicted well both the ‘metabolic memory’ phenomenon and the detrimental effects of unstable glucose. With only a few parameters, it reproduced complex relationships between glucose dynamics and oxidative stress, and allowed us to identify underlying drivers of the observed phenomenon. Further work may include model updating and calibration for *in vivo* conditions, which, together with daily plasma glucose profiles, would allow us to predict oxidative stress levels and their link to the potential risk of complications in diabetic patients.
